# Pelargonidin ameliorates MCAO-induced cerebral ischemia/reperfusion injury in rats by the action on the Nrf2/HO-1 pathway

**DOI:** 10.1515/tnsci-2021-0006

**Published:** 2021-01-12

**Authors:** Kong Fu, Miancong Chen, Hua Zheng, Chuanzi Li, Fan Yang, Qian Niu

**Affiliations:** Department of Radiology, The Second Affiliated Hospital, Hainan Medical University, Haikou, 570311, China; Department of Critical Care Medicine, The First Affiliated Hospital of Hainan Medical University, Haikou, 570102, China; Office of Acupuncture Clinical, College of Traditional Chinese Medicine, Hainan Medical University, No. 3, Xueyuan Road, Longhua District, Haikou, 571199, China

**Keywords:** Nrf2/HO-1 pathway, MCAO, pelargonidin, stroke, cerebral ischemia/reperfusion

## Abstract

**Background:**

Morbidity and mortality remain high for ischemic stroke victims, and at present these patients lack effective neuroprotective agents, which improve the cure rate. In recent years, studies have shown that pelargonidin has many biological actions. However, few studies are available regarding the pelargonidin treatment of cerebral ischemia.

**Methods:**

The rat middle cerebral artery occlusion (MCAO) model was established to investigate the neuroprotective effect of pelargonidin on cerebral ischemia/reperfusion injury. Reperfusion was performed 2 h after ischemia; magnetic resonance imaging (MRI) and 2, 3, 5-triphenyltetrazolium chloride (TTC) staining were used to measure the volume of cerebral ischemia. Both modified neurological severity scores (mNSSs) and Morris water maze test were used to assess the neurological functions. ELISA was applied to determine the levels of TNF-α, TGF-β, IL-6, IL-10, MDA, and SOD. The expression of Nuclear factor-E2-related factor 2 (Nrf2) and heme oxygenase 1 (HO-1) protein in brain tissue was measured by immunofluorescence and Western blot assays.

**Results:**

The results showed that pelargonidin could effectively reduce the volume of cerebral ischemia and improve the neurological function in MCAO rats, thereby improving memory and learning ability. With the corresponding decreases in the expression of TNF-α, TGF-β, IL-6, and MDA, the level of IL-10 and SOD increased and also promoted the nuclear metastasis of Nrf2 and the expression of HO-1 in ischemic brain tissues.

**Conclusions:**

Our data demonstrated that pelargonidin ameliorated neurological function deficits in MCAO rats, and its potential mechanism of action was associated with overexpression of the Nrf2/HO-1-signaling pathway. This study will provide a new approach to treat cerebral ischemia/reperfusion injury.

## 1 Background

Stroke is a group of diseases associated with sudden rupture of cerebral vessels or brain tissue injury caused by blockage of blood flow to the brain and is characterized by high morbidity, mortality, and disability rates. According to epidemiological studies, approximately 80.1 million people suffer from stroke worldwide, of which 41.1 million are female and 39 million are male; in 2016, 13.7 million patients were newly diagnosed with stroke [1]. Atherosclerotic disease is the main causative factor for stroke. Patients with dyslipidemia and/or diabetes mellitus are more likely to suffer stroke [2,3]. Stroke can be classified into two types: ischemic and hemorrhagic, with about 60–80% of strokes being ischemic in nature [4]. The brain functions, such as locomotor function, memory, thinking, and language, are greatly impaired after stroke [2]. Timely reperfusion is an effective method to treat stroke. However, reperfusion could also induce additional impairments to neurological functions [5]. Although recombinant tissue-type plasminogen activator (r-tPA) is currently the most effective way to restore blood supply in ischemic stroke, only about 3–5% of ischemic stroke patients are effectively treated due to a narrow time window for r-tPA treatment [6,7,8]. Patients with ischemic stroke may benefit from neuroprotective agents in the subacute phase or the late stage of blood flow restoration [5]. Therefore, it is important to find effective neuroprotective agents that can successfully treat ischemic/reperfusion injury.

The activation of inflammatory cells and increased pro-inflammatory factors, combined with oxidative stress and free radical generation, induce neuron apoptosis, axon degeneration, synaptic plasticity, and transmission impairment [9,10,11]. Nuclear factor-E2-related factor 2 (Nrf2), as an endogenous factor in brain tissue and a member of the leucine zipper family of transcription factors, plays an important role in reducing oxidative stress and inflammation [12,13]. In response to cellular oxidative damage, activated Nrf2 translocates into nucleus and binds to the promotor regions of antioxidant response genes, thus regulating the expression of the downstream antioxidant genes, such as enzyme heme oxygenase 1 (HO-1). HO-1 and its enzymatic products possess antioxidant, anti-inflammatory, antiapoptotic and vasodilation actions, with concomitant improvement in the tissue microcirculation [14,15]. Recently, it has been shown that Nrf2 activation attenuated oxidative damage induced by cerebral ischemic injury and that HO-1-deficient mice exhibited more severe brain injury [16,17]. As a result, the Nrf2/HO-1 pathway may be considered as a potential target for neuroprotective therapy in ischemic brain injury.

**Figure 1:**
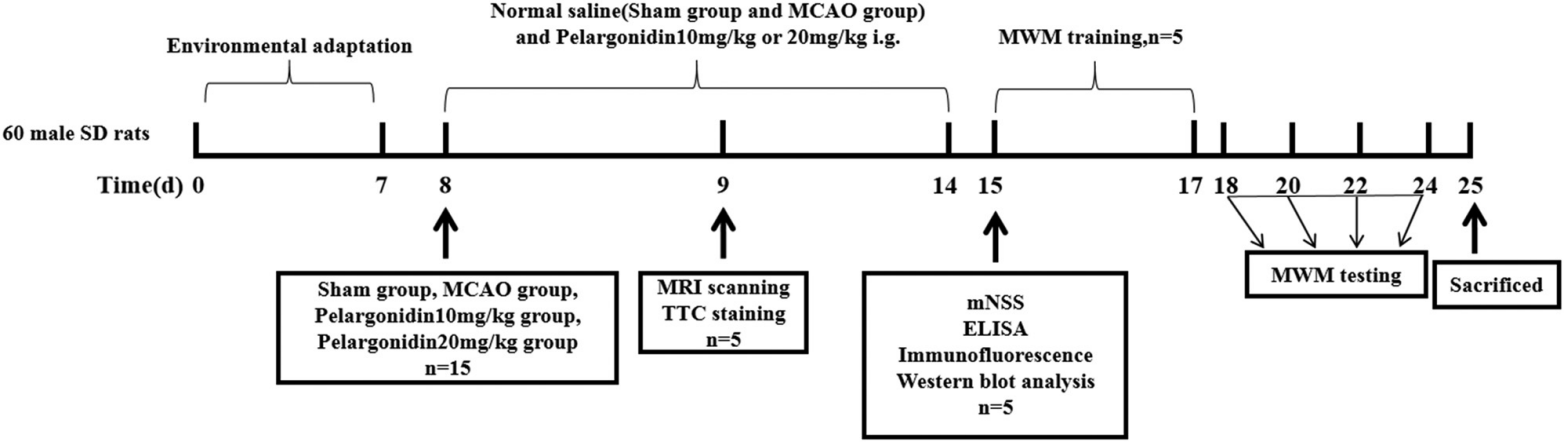
Flowchart of the MCAO experiments.

Evidences have proved many drugs could protect the neurological functions after stroke. *N*-Palmitoylethanolamide-oxazoline could decrease brain ischemic/reperfusion injury via targeting the Sirtuin 1 (SIRT1) pathway [2]. Coenzyme Q10 single intravenous injection could limit brain ischemia damage [18]. Anthocyanidins have potent antioxidant and anti-inflammatory effects. As a member anthocyanidin, pelargonidin (Pel, chemical formula shown in Figure 1a) is widely distributed in vegetables and fruits, e.g., carrots, berries, blueberries, strawberries, and pomegranates [19,20]. Several studies have demonstrated that pelargonidin possess antioxidant [21], anti-inflammatory [22], antithrombotic [23], and antidiabetic [24]functions. In addition, pelargonidin can ameliorate memory impairment in a rat model of Alzheimer’s disease by inhibiting glial activation and oxidative stress [25]. However, the potential biological activities and mechanism of pelargonidin as an antioxidant and anti-inflammatory factor in cerebral ischemia/reperfusion (I/R) injury remain unclear.

In the present study, the rat middle cerebral artery occlusion (MCAO) model was used to investigate the neuroprotective effect and potential mechanisms of pelargonidin on cerebral I/R injury. Our study showed that pelargonidin could effectively reduce the infarct area, improve neurological functions, significantly reduce the level of inflammatory and oxidative factors, and promote the repair of neuronal cells in brain tissue after cerebral I/R. The neuroprotective effect of pelargonidin on cerebral I/R injury was associated with overexpression of the Nrf2/HO-1 pathway.

## 2 Materials and methods

### 2.1 Animals

Male Sprague-Dawley (SD) rats (220–260 g) were purchased from Shanghai Alac Laboratory Animal Co. Ltd. (Shanghai, China); License No.: SCXK (Shanghai) 2017-0005 and Certificate No.: 20170005008495. All rats were fed with a standard rodent diet, sterilized secondary ultrapure water *ad libitum*, housed at 22–25°C with a humidity of 40–70% in a 12-h light–dark cycle. The animals were left to acclimatize for 7 days.


**Ethical approval**: The research related to animals’ use has been complied with all the relevant national regulations and institutional policies for the care and use of animals.

### 2.2 Group assignment and drug administration

A total of 60 SD rats were randomly divided into four groups (*n* = 15): sham, MCAO, MCAO + Pel 10 mg/kg, and MCAO + Pel 20 mg/kg. Pelargonidin (Chengdu Herbpurify Co., Ltd., Chengdu, China; EINECS No.: 205-127-7, purity ≥98%) was dissolved in sterile distilled water. Rats in the experimental groups were orally administered 10 or 20 mg/kg of pelargonidin per day [26,27], while the sham and MCAO groups were given the same volume of the normal saline daily for 7 days (Figure 1).

### 2.3 MCAO model

The rat model of focal cerebral I/R injury was prepared using the suture-occluded method developed by Longa. The rats were anesthetized via intraperitoneal injection of 10% chloral hydrate (Sigma-Aldrich, CA, USA; 300 mg/kg) and then fixed in the supine position. The neck was shaved and disinfected for routine skin preparation. A midline cervical incision was made to dissect the right common carotid, external and internal carotid arteries, followed by ligation of the external carotid artery and also its distal end. The proximal ends of the common carotid and internal carotid arteries were temporarily clipped. A small incision was made in the external carotid artery adjacent to the common carotid bifurcation and a silicon-coated suture was inserted. The clip over the internal carotid artery was removed and the suture was gently inserted into the internal carotid artery through the external carotid artery until the origin of the middle cerebral artery was occluded. The length of the suture was 18–20 mm. The suture was tightened and the clip over the common carotid artery removed. Then the cervical skin was sutured; 2 h after the ischemia status, the suture was removed, allowing reperfusion of the blood supply. In the sham group, only the internal carotid artery was dissected without any other procedures. After surgery, 100,000 units of penicillin sodium (Sigma-Aldrich, CA, USA) were injected intramuscularly for 3 consecutive days to prevent infection.

### 2.4 Magnetic resonance image (MRI) scanning

After treatment, the rats were examined using MRI scanning (GE Discovery MR750W 3.0T Superconducting MRI System) with a 3T experimental coil (5 cm in aperture). The rats were anesthetized by intraperitoneal injection of 10% chloral hydrate (300 mg/kg) and fixed in the supine position, with the head placed through the coil centrally. T2-weighted MRI scan (T2WI) and coronal diffusion-weighted imaging (DWI) were performed. An echo planar imaging sequence was obtained for DWI with TR = 350 ms, TE = 50 ms, *b* value = 1,000 s/mm^2^, slice thickness = 3 mm, and slice gap = 0.2 mm. The images were processed by Functool software, and the largest slice of ischemic lesions was selected for analysis. Region of interest (ROI) with an area of 2 mm^2^ was placed in the lesion center and contralateral mirror location. The relative apparent diffusion coefficient (rADC) and exponential ADC (eADC) signals of both lesion side and mirror normal side were measured. rADC and reADC, the relative values of the lesion side vs the mirror normal side (rADC = ipsilateral ADC value/homologous contralateral ADC value and reADC = ipsilateral eADC value/homologous contralateral eADC value), were calculated.

### 2.5 Neurological function tests (modified neurological severity score [mNSS])

On day 2 after the end of treatment, the mNSS was used to evaluate the neurological functions of MCAO rats in each group; mNSS was a scale with a total score of 18 and evaluates motor, sensory, reflex, and balance functions. The function was considered normal when the mNSS was 0, and a higher mean mNSS indicated a higher severity of neurological impairment [22]. The scoring details are shown in Table S1.

### 2.6 2,3,5-Triphenyltetrazolium chloride (TTC) staining

Rats were anesthetized by intraperitoneal injection of 10% chloral hydrate (300 mg/kg) and then the thoracic cavity and right auricular appendix were opened. They were then transcardially perfused with PBS and then the whole brain was removed and sectioned into five slices. The sections were incubated in 2% TTC (Sigma-Aldrich, CA, USA) at 37°C for 30 min, fixed in 4% formaldehyde for 24 h, and then photographed. Additionally, the infarct size was calculated.

### 2.7 Morris water maze (MWM) testing

The MWM test was performed to assess the spatial learning and memory of the rats. A round pool (120 cm diameter, 50 cm height, 30 cm depth, and water temperature 22 ± 1°C) was placed in an independent light-protected laboratory house and divided into four quadrants (E: East, S: South, W: West, and N: North). Each rat was trained twice daily before the MWM at 120 s/dose for 3 days at the end of the treatment. On day 18, 20, 22, and 24, the place navigation test was performed. The platform was placed in any quadrant 2 cm under water. The adjacent and opposite quadrants of the platform were selected as water entry points. The latency and times of crossing the platform were measured during a 120-s test. The assay was performed according to the instructions of the instrument supplier (SuperMaze MWM Experimental Analysis System, Shanghai XinRuan Information Technology Co., Ltd.).

#### 2.7.1 ELISA

Blood was collected from the abdominal inferior vena cava. After placing at room temperature for 2 h, the blood samples were centrifuged at 3,000 rpm for 10 min at 4°C to separate the serum. The levels of TNF-α, TGF-β, IL-6, IL-10, MDA, and SOD in rat blood serum were measured with ELISA kit (R & D Systems, Minneapolis, MN, USA).

### 2.8 Immunofluorescence

Rats were anesthetized by intraperitoneal injection of 10% chloral hydrate (300 mg/kg) and decapitated to facilitate the removal of their brains. Brain tissues were fixed in 4% paraformaldehyde for 72 h, embedded in paraffin, and sectioned (4 μm slices). The sections were dehydrated with gradient alcohol and washed 3 times with PBS. They were blocked with 10% fetal bovine serum (Gibco Life Technologies, NY, USA) for 2 h and then incubated with anti-Nrf2 antibody and anti-HO-1 antibody (1:100; Abcam, Cambridge, MA, USA) at 4°C overnight, followed by washing three times with PBS. Fluorescence (red light)-conjugated secondary antibody IgG (1:200; MultiSciences, Shanghai, China) was added and incubated for 2 h at room temperature, followed by washing three times with PBS. The slides were counterstained with DAPI (Beyotime Biotechnology, Shanghai, China) for 10 min and photographed under a fluorescence microscope.

### 2.9 Western blot analysis

The ischemic cortex tissues were isolated and homogenized using RIPA lysate (Beyotime Biotechnology) until no obvious lump was observed. Tissues were then centrifuged at 14,000 rpm for 30 min at 4°C and the supernatant was collected to obtain the total protein. To detect the protein expression in nuclear, the Nuclear-Cytosol Extraction kit (Beyotime Biotechnology) was applied. The cortex tissues were homogenized with cold PBS until no obvious lump. The supernatant was collected and centrifuged (4℃, 500 *g* × 3 min). The supernatant was discarded, and the separation buffer and proteinase inhibitor were added. After centrifuging (4℃, 16,000 *g* × 5 min), the cytoplasmic proteins in the supernatant were collected, while the nuclear proteins in the sediment were also collected. The protein concentration of samples was determined with a BCA Protein Assay kit (Beyotime Biotechnology). The protein of samples were electrophoresed with 10% SDS-PAGE and transferred onto PVDF membranes (Millipore, MA, USA), The membranes were blocked with 5% skimmed milk for 2 h and washed three times in TBST-buffered saline. Next they were incubated with Nrf2 and HO-1 (1:1,000; Abcam) monoclonal antibodies at 4°C overnight. Subsequently, the membranes were washed three times with TBST-buffered saline, incubated with anti-IgG antibody (1:2,000; MultiSciences, Shanghai, China) at room temperature for 1 h, and washed three times with TBST-buffered saline. The ECL Chemiluminescent kit (Beyotime Biotechnology) was used in a dark room for gel-imaging acquisition and analysis. Quantity One software was used to analyze the corresponding gray scale values of each band.

### 2.10 Data analysis

Data were collected from five rats separately in each experiment for statistical analysis. For each experiment, the data were expressed as the mean ± SEM and were statistically analyzed using GraphPad Prism 6.0 software. Statistical differences between data were assessed using one-way ANOVA followed by the Newman–Keuls test. A *P* value < 0.05 was considered to be a statistically significant finding.

## 3 Results

### 3.1 Pelargonidin reduces the cerebral ischemic area in MCAO rats

**Figure 2:**
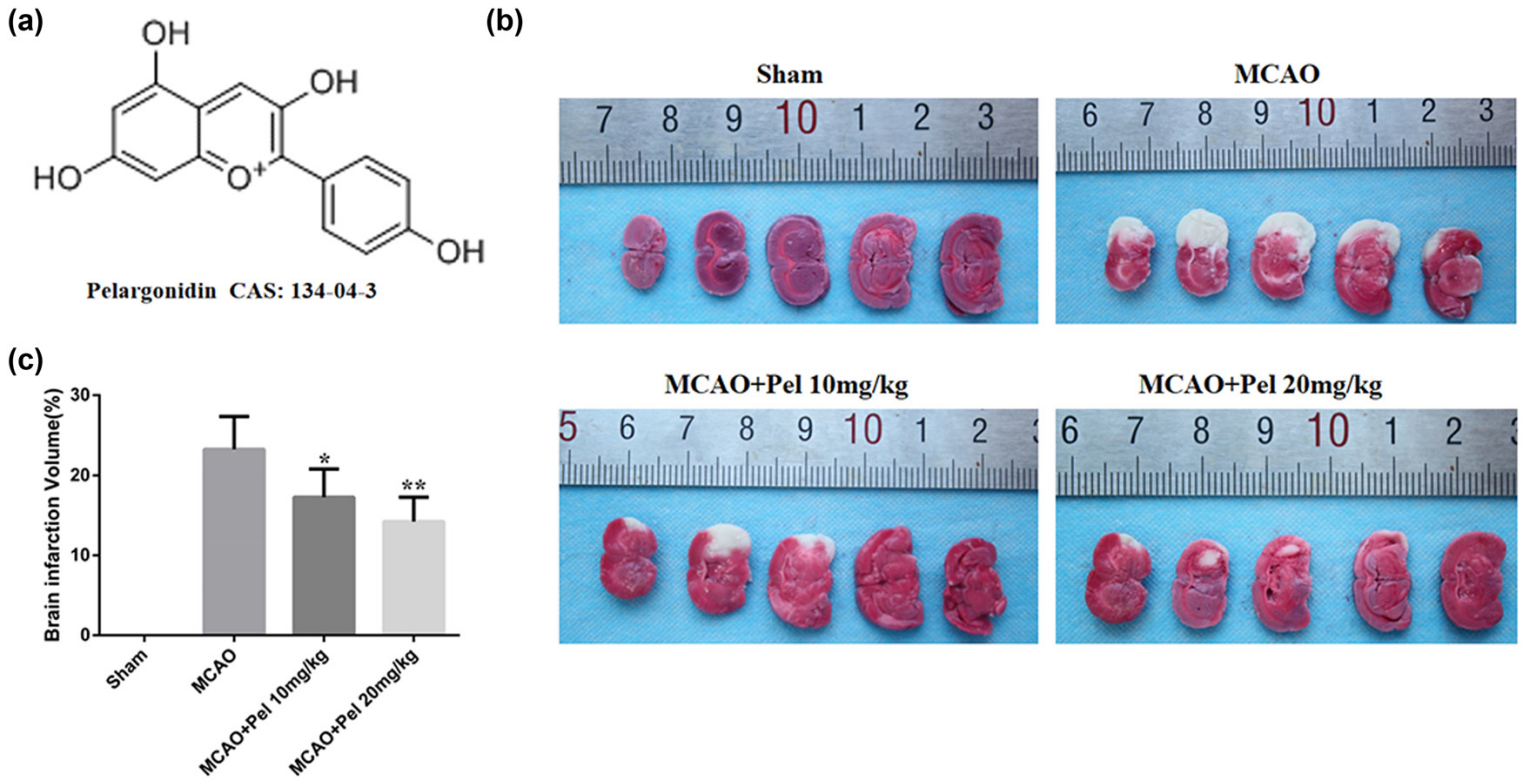
Cerebral infarction volume of rats in each group was compared. (a) Pelargonidin chemical formula and Chemical Abstracts Service (CAS) number. (b) TTC staining of typical brains of experimental rats. (c) Cerebral ischemic volume of experimental rats in each group (*n* = 5). ^*^ *P* < 0.05 and ^**^ *P* < 0.01 compared with MCAO group.

**Figure 3:**
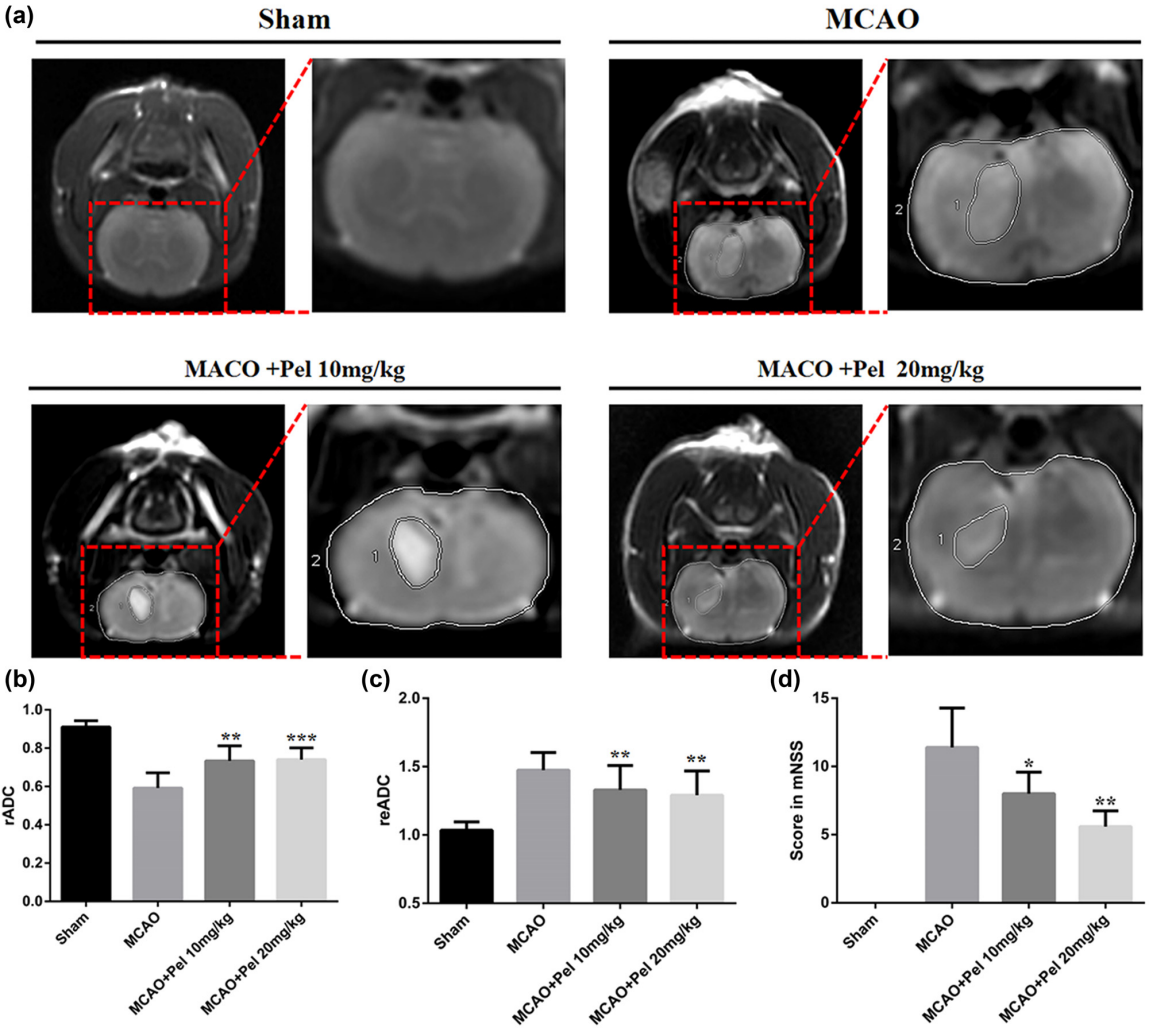
MRI detection of cerebral ischemia in each group of experimental rats. (a) Typical images of coronary angiography of T2WI scans of experimental rats in each group. (b and c) The rADC value and reADC value of experimental rats in each group (*n* = 5). (d) The mNSSs was used to evaluate the effect of pelargonidin on the neural function of rats in each experimental group (*n* = 5). ^*^ *P* < 0.05, ^**^ *P* < 0.01, and ^***^ *P* < 0.001 compared with the MCAO group.

MRI and TTC staining were used to measure the extent of cerebral infarction in MCAO rats treated with pelargonidin. Figure 2b and c showed that TTC staining failed to reveal any infarct areas in the sham group, but there was an obvious white infarct area in the MCAO group (23.30 ± 4.10). The infarct areas in the pelargonidin 10 mg/kg (17.29 ± 3.52) and 20 mg/kg (14.24 ± 3.04) groups were significantly reduced compared to that in the MCAO group (*P* < 0.05). On MRI, the T2WI sequence revealed no abnormalities in the bilateral cerebral hemispheres of the sham rats, but there was an abnormal signal intensity in the MCAO group. Rats in both the pelargonidin 10 mg/kg and 20 mg/kg groups had significantly fewer infarct lesions compared to that in the MCAO group, which exhibited enlarged infarct lesions in brain tissue. The rADC values for both the MCAO and pelargonidin-treated rats were significantly lower than those in the sham group. The rADC values in the pelargonidin 10 mg/kg and 20 mg/kg groups were higher than those in the MCAO group. Although rats in both the MCAO and pelargonidin-treatment groups had higher reADC values than rats in the sham group, the reADC values in both the pelargonidin 10 mg/kg group and pelargonidin 20 mg/kg group were lower than those in the MCAO group (Figure 3a–c).

### 3.2 Pelargonidin improves neurological functions in MCAO rats

**Figure 4:**
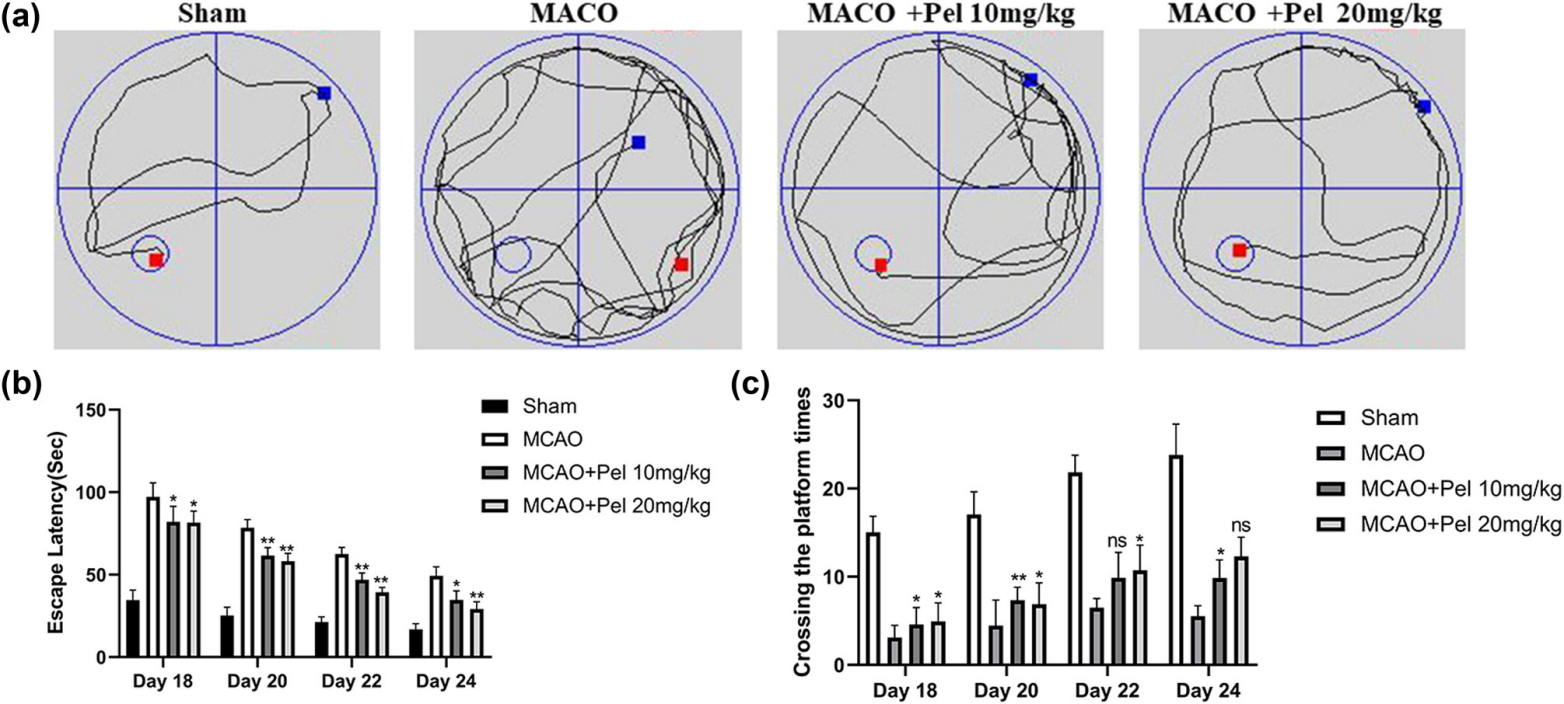
MWM was used to detect spatial learning and memory in rats. (a) Motion trajectory diagram of MCAO rats in MWM. (b and c) The escape latency and number of times each group crosses the platform within 2 minutes (*n* = 5). ^*^ *P* < 0.05 and ^**^ *P* < 0.01 compared with MCAO group, ns: no significance.

We used mNSS to assess the recovery of neurological functions in the MCAO rats (a higher mNSSs indicated less recovery). As shown in Figure 3d, the mean mNSSs in the sham group was 0, indicating normal neurological functions. The mean mNSSs in the MCAO group and pelargonidin-treatment groups increased compared with the sham group. However, both of the pelargonidin groups had a lower mean mNSSs than the MCAO group (*P* < 0.05). Besides, the MWM test was performed to analyze the spatial learning and memory capabilities of the rats. A 4-day MWM test demonstrated that the latency to escape was shortened for all MCAO rats (Figure 4a–c). However, the latency to escape in both 10 and 20 mg/kg the pelargonidin groups was significantly shorter than that in the MCAO for the different 4-day tests. Similarly, the rats in the pelargonidin 10 and 20 mg/kg groups crossed the platform more frequently than rats in the MCAO group during the different 4-day tests. These findings suggested that pelargonidin could partially improve the neurological functions of I/R rats and thus enhance their memory and learning capabilities.

#### 3.2.1 Pelargonidin reduces the levels of inflammatory factors in the brains of MCAO rats and exerts antioxidative effects

**Figure 5:**
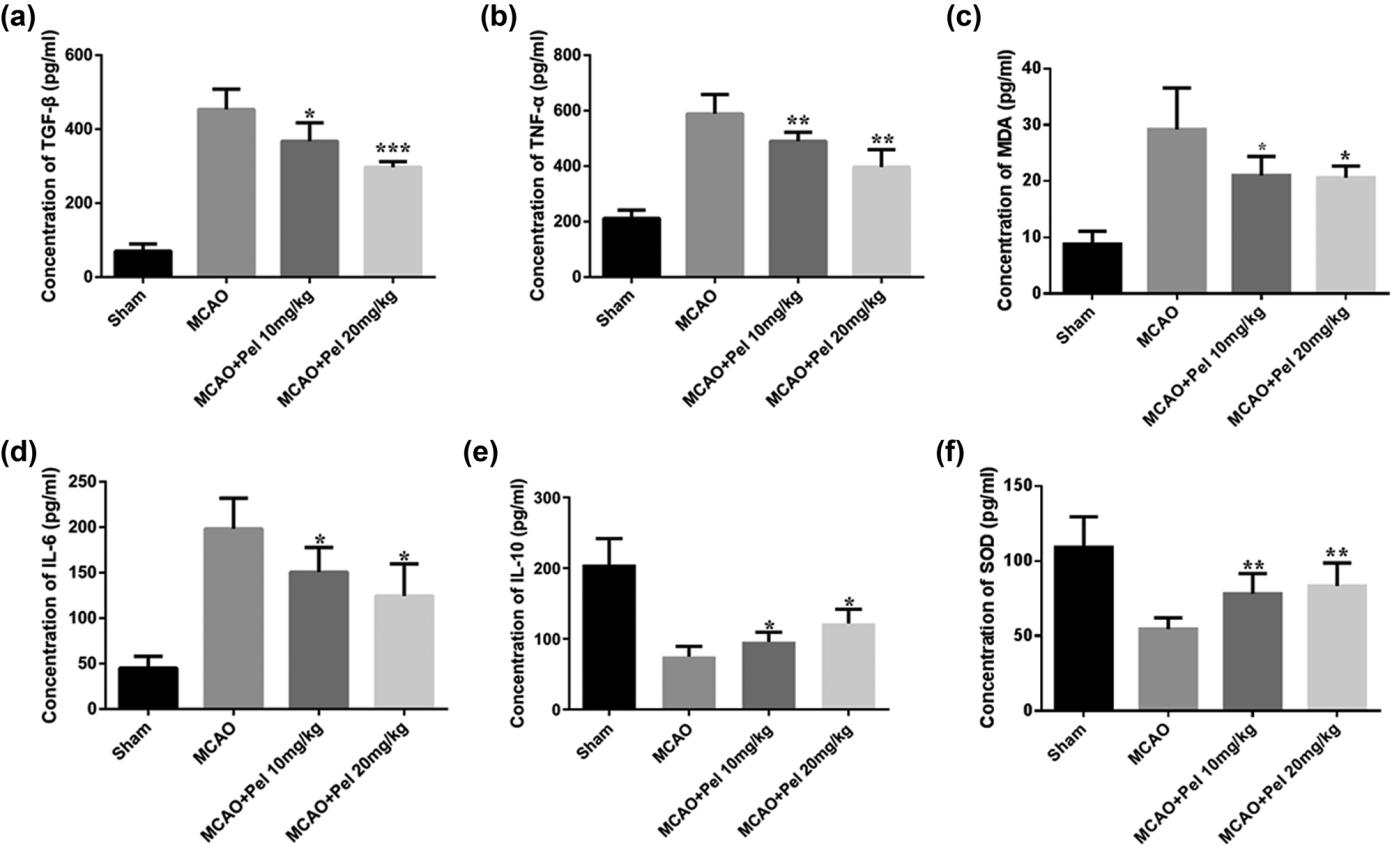
Serum levels of inflammatory factors (TNF-α, TGF-β, IL-6, and IL-10) and oxidative factors (MDA and SOD) in rats of all experimental groups were determined by ELISA (*n* = 5). ^*^ *P* < 0.05, ^**^ *P* < 0.01, and ^***^ *P* < 0.001 compared with the MCAO group.

The pathophysiological mechanism underlying cerebral I/R injury is very complex. Such injuries are commonly caused by the release of inflammatory factors and oxidative damage. Hence, ELISA was used to measure the levels of TGF-β, TNF-α, IL-6, IL-10, MDA, and SOD in samples of rat blood serum. Figure 5a–f showed that the levels of TGF-β, TNF-α, IL-6, and MDA in the MACO group were higher compared to those in the sham group as well as the levels in both the pelargonidin 10 and 20 mg/kg groups (*P* < 0.05). The serum levels of IL-10 and SOD in the sham group were higher than those in the MACO group as well as those in the pelargonidin 10 and 20 mg/kg groups (*P* < 0.05). The above results suggested that pelargonidin attenuated inflammatory responses and the degree of oxidative damage in brain tissues in MCAO-induced rats.

#### 3.2.2 Pelargonidin exerts neuroprotective effects by activating the Nrf2/HO-1 pathway

**Figure 6:**
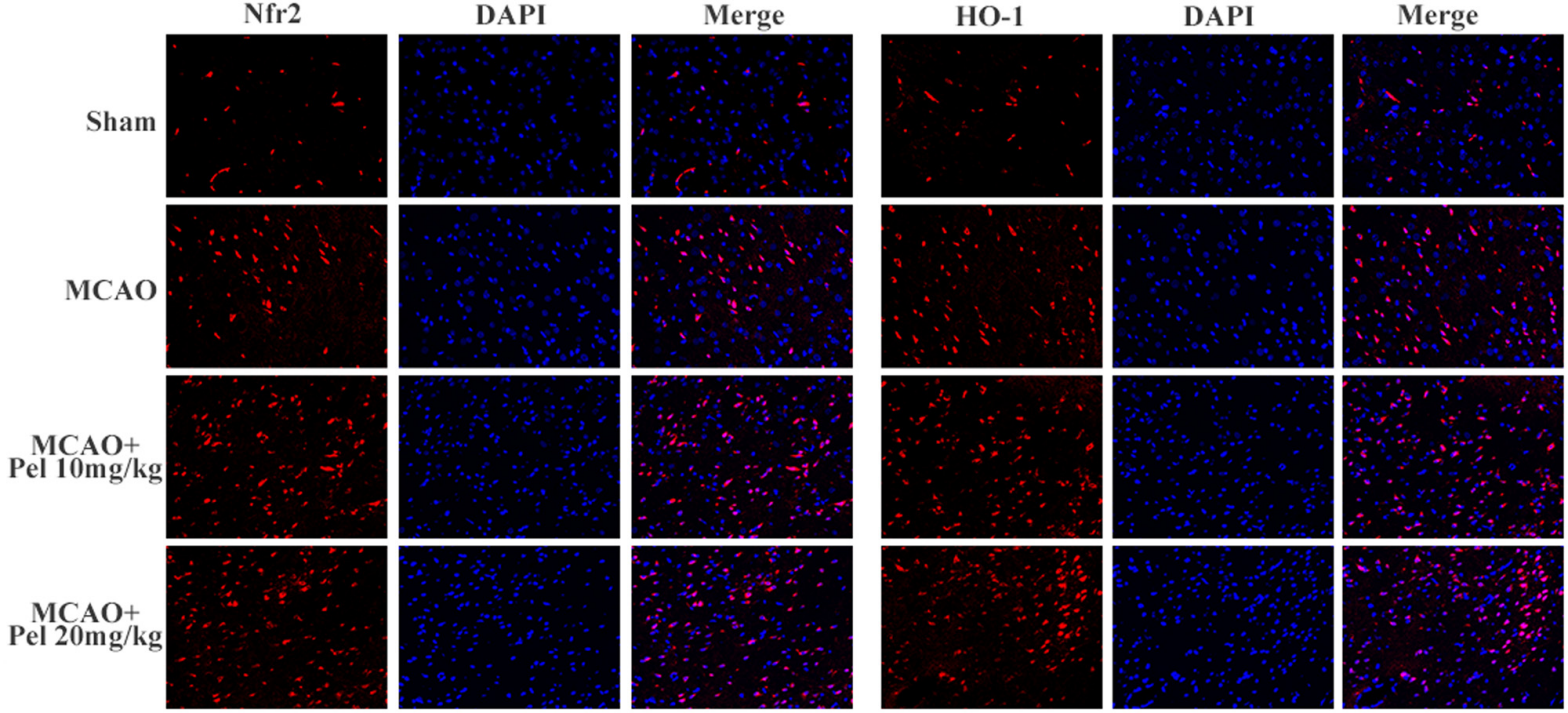
Immunofluorescence was used to observe the expression of Nrf2 and HO-1 in the brain tissues of rats in each group (scale bar = 100 μm).

**Figure 7:**
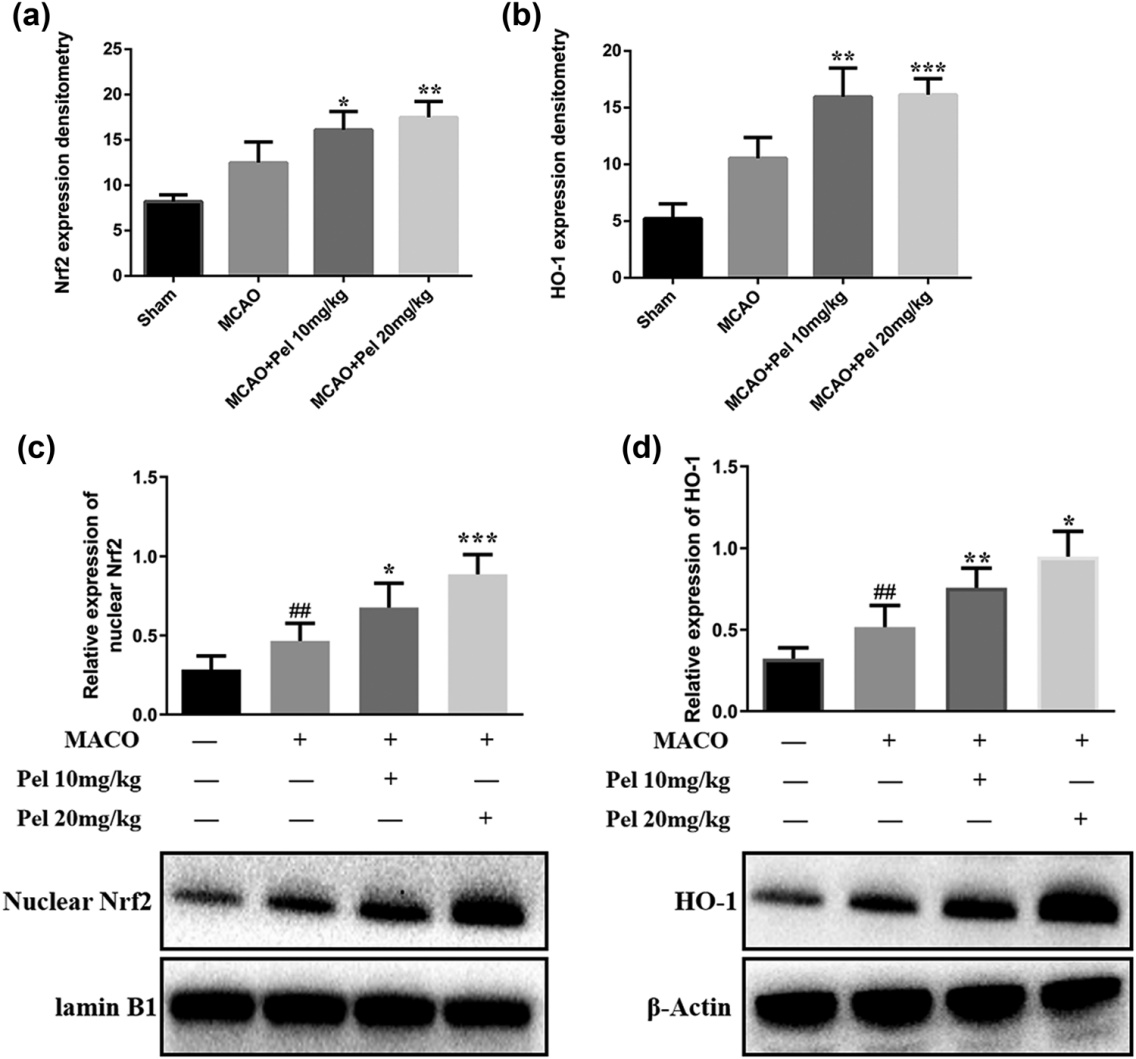
Expression levels of Nrf2 and HO-1 in rat brain tissue. (a and b) The relative expression of Nrf2 and HO-1 in rat brain tissue was detected by immunofluorescence (*n* = 5). (c and d) The relative expressions of Nrf2 and HO-1 in brain tissues of rats in each group were determined by Western blot (*n* = 5). ^*^ *P* < 0.05, ^**^ *P* < 0.01, and ^***^ *P* < 0.001 compared with MCAO group; ^##^ *P* < 0.01 compared with the sham group.

To further examine the mechanism of pelargonidin protecting brain tissues against MACO-induced I/R injuries, the expression of Nrf2 and HO-1 proteins (components of the Nrf2/HO-1 pathway) in the infarcted brain tissues was detected by immunofluorescence and Western blot assays. The results revealed that the levels of nuclear metastasis of Nrf2 and the expression of HO-1 in the MCAO group were higher than those in the sham group but lower than that in either the pelargonidin 10 mg/kg group or the pelargonidin 20 mg/kg group (*P* < 0.05; Figures 6 and 7a–d). These findings indicated that the neuroprotection effect of pelargonidin was accompanied by the overexpression of the Nrf2/HO-1 pathway.

## 4 Discussion

The rat MCAO-induced cerebral I/R injury rat model is frequently used for cerebral ischemia studies, because its pathophysiological changes and gene expression alterations are similar to those in humans [2]. The determination of infarct volume and a behavioral evaluation of neurological functions are important factors for measuring the effect of any treatment of cerebral ischemia [28].

When cerebral ischemia develops at the reperfusion stage, the compensation provided by the preexisting nerve cells in the ischemic and hypoxic brain tissues rises abruptly and is accompanied by a dramatic increase in free radicals that mediate oxidative damage in the affected areas [29,30]. As an end product of oxidation, MDA further aggravates any damage to cellular membranes. Because SOD is the leading scavenger of free radicals, the severity of oxidative damage in a cerebral ischemic area will depend on the balance between MDA and SOD levels [31,32]. On the other hand, oxygen free radicals and other messengers that reside in the ischemic area will help to upregulate the production of adhesion molecules; this allows leukocytes to accumulate in microvessels and ultimately creates a vascular obstruction. During this process, inflammatory factors such as TGF-β, TNF-α, and IL-6 may also be produced. In addition to an increased infiltration of inflammatory cells, these factors also contribute to the extracellular release of numerous inflammatory mediators, which further aggravate damage to the ischemic area [33,34].

The Nrf2/HO-1 pathway plays an important role in preventing oxidative stress *in vitro* and *in vivo* [35]. Under physiological conditions, Nrf2 is predominantly retained in the cytoplasm by forming a complex with Keap 1. When exposed to external stress, such as ROS, the Nrf2 inducer reacts with Keap1 cysteine to release Nrf2 protein, which subsequently translocates to the cell nucleus, where it activates Nrf2 and downstream antioxidant enzymes [17]. As a transcription factor, nuclear Nrf2 induces many target gene transcription, such as HO-1, NADPH, GST, and thioredoxin [36]. Nrf2 could exert both antioxidant and anti-inflammatory roles [37,38,39]. The activation of Nrf2/HO-1 pathway exerts a neuroprotective role in Parkinson’s disease [40]. The inflammation-related pathway, NF-κB, and nucleotide-binding oligomerization domain, leucine rich repeat and pyrin domain containing 3 (NLRP3) inflammasome could also be modulated by Nrf2 [40]. The lipid peroxidation, oxidative stress, and inflammation could be inhibited by hydroxytyrosol via upregulating Nrf2 in the pancreatitis-associated gut injury [36]. HO-1 is an inducible rate-limiting enzyme for heme catabolism in the microsomal enzyme system. As an antioxidant enzyme targeting Nrf2, HO-1 is essential for preventing cerebral ischemic injuries, Parkinson’s disease, and other neurodegenerative disorders [41,42].

Pelargonidin is an extracted nature component from ripe raspberries, strawberries, and blueberries [43]. Pelargonidin has been shown to have many biological roles, including antioxidation, anti-inflammation, antidiabetic, and antithrombotic functions [21,22,23,24]. It could protect many types of cells by increasing detoxification enzymes to inhibit ROS generation [21,43]. Pelargonidin could prevent liver fibrosis via Nrf2-inhibited ROS/NLRP3/IL-1β axis and inhibit inflammasome-related genes and IL-1β secretion in a dose-dependent manner [44]. It has previously been shown that pelargonidin could significantly attenuate MDA and catalase activity in the hippocampus, decrease glial fibrillary acidic protein (GFAP) levels, and thereby improve memory and learning functions in a rat model of Alzheimer’s disease [25]. Pelargonidin has been found to inhibit transforming growth factor–beta–induced protein (TGFBIp)-induced human umbilical vein endothelial cell hyperpermeability, the expression of cell adhesion molecules, and the adhesion and migration of leukocytes [45]. It also inhibited the LPS-induced inflammatory response; helped to reduce the expression of TNF-α, IL-6, NF-κB, and other factors; and decreased mortality due to LPS-induced endotoxemia in mice [46].

In the present study, both MRI and TTC staining results revealed that pelargonidin could effectively reduce the brain infarct volume of I/R rats. Pelargonidin could also enhance the performance of motor activity, sensory skills, reflexes, and balance of MCAO rats. Further, pelargonidin could also improve their memory and learning abilities. The above results also suggested that pelargonidin can improve the neurological functions of MCAO rats and may be a candidate for development as a neuroprotective agent for the treatment of cerebral I/R injuries. We found that pelargonidin could decrease the levels of MDA (an oxidative factor), TNF-α, TGF-β, and IL-6 in the serum of MCAO rats and elevate the expression of SOD (an antioxidative factor) and IL-10 (an anti-inflammatory factor). Our results suggest that pelargonidin can attenuate oxidative stress and inflammatory responses that occur in an MCAO-induced rat model of I/R.

Pelargonidin has been shown to reduce TPA-induced methylation of the *Nrf2* gene promoter region in murine epidermal JB6 cells and to enhance the expression of HO-1 (a downstream target gene for Nrf2), and thereby helps to provide cytoprotection [43]. It has also been reported that pelargonidin upregulates the Keap1/Nrf2-signaling pathway and ameliorates citrinin-induced oxidative stress injuries in HepG2 cells [21]. Our research results show that after cerebral I/R injury, the nuclear metastasis of Nrf2 and expression of HO-1 increased in rat brain tissue. It is suggested that although cerebral I/R injury can promote the nuclear metastasis of Nrf2 and the expression of HO-1, its protective effect on neurological functions is also limited. When we applied pelargonidin, the nuclear metastasis of Nrf2 and expression of HO-1 significantly increased compared to the MCAO group, indicating that pelargonidin can promote the effect of Nrf2/HO-1 to neuroprotective.

## 5 Conclusions

The present study has demonstrated that pelargonidin attenuated oxidative stress and inflammatory damage in the cerebral I/R tissues of rats and thereby exerted neuroprotective effects. Our results also showed that the effects of pelargonidin were achieved by inducing overexpression of the Nrf2/HO-1 pathway. These findings provide a new perspective on the development of agents for protecting against cerebral ischemia and cerebral I/R injuries.
